# Central venous access related adverse events after trabectedin infusions in soft tissue sarcoma patients; experience and management in a nationwide multi-center study

**DOI:** 10.1186/s13569-017-0066-6

**Published:** 2017-01-31

**Authors:** Michiel C. Verboom, Jan Ouwerkerk, Neeltje Steeghs, Jacob Lutjeboer, J. Martijn Kerst, Winette T. A. van der Graaf, Anna K. L. Reyners, Stefan Sleijfer, Hans Gelderblom

**Affiliations:** 1 Department of Medical Oncology, Leiden University Medical Center, Postbus 9600, 2300 RC Leiden, The Netherlands; 2 Department of Medical Oncology, Antoni van Leeuwenhoek-Netherlands Cancer Institute, Postbus 90203, 1006 BE Amsterdam, The Netherlands; 3 Department of Radiology, Interventional Radiology Section, Leiden University Medical Center, Postbus 9600, 2300 RC Leiden, The Netherlands; 4 Department of Medical Oncology, Radboud University Medical Center, Postbus 9101, 6500 HB Nijmegen, The Netherlands; 5 The Institute of Cancer Research and the Royal Marsden NHS Foundation Trust, 123 Old Brompton Road, London, SW7 3RP UK; 6 Department of Medical Oncology, University of Groningen, University Medical Center Groningen, Postbus 30.001, 9700 RB Groningen, The Netherlands; 7 Department of Medical Oncology, Erasmus MC Cancer Institute, Postbus 2040, 3000 CA Rotterdam, The Netherlands

**Keywords:** Trabectedin, Central venous catheters, Adverse events, Soft tissue sarcoma

## Abstract

**Background:**

Trabectedin has shown efficacy against soft tissue sarcomas (STS) and has manageable toxicity. Trabectedin is administered through central venous access devices (VAD), such as subcutaneous ports with tunneled catheters, Hickman catheters and PICC lines. Venous access related adverse events are common, but have not yet been reported in detail.

**Methods:**

A retrospective analysis of patient files of STS patients receiving trabectedin monotherapy between 1999 and 2014 was performed in all five STS referral centers in the Netherlands. This survey focused on adverse events related to the VAD and the actions taken in response to these events.

**Results:**

In the 127 patients included in this analysis, 102 venous access ports (VAP), 15 Hickman catheters and 10 PICC lines were used as primary means of central venous access. The most frequently reported adverse events at the VAD site were erythema (30.7%), pain (28.3%), inflammation (11.8%) and thrombosis (11.0%). Actions taken towards these adverse events include oral antibiotics (17.3%), VAD replacement (15.0%) or a wait-and-see policy (13.4%). In total, 45 patients (35.4%) with a subcutaneous port developed a varying degree of inflammation along the trajectory of the tunneled catheter. In all but three patients, this was a sterile inflammation, which was considered a unique phenomenon for trabectedin. Microscopic leakage of trabectedin along the venous access device and catheter was considered the most plausible cause for this adverse event. Placing the catheter deeper under the skin resolved the issue almost completely.

**Conclusion:**

Trabectedin infusion commonly leads to central venous access related adverse events. Sterile inflammation along the catheter trajectory is one of the most common adverse events and can be prevented by placing the catheter deeper under the skin.

## Background

Cytostatic drugs infused directly into peripheral veins can have very damaging effects on these blood vessels. To ensure safe and durable administration of such agents, several methods have been developed in the past, like the arteriovenous shunt, which is no longer used for the infusion of chemotherapy [1]. In 1982, a central venous access device was introduced, that used a subcutaneous reservoir and a tunneled catheter to provide access to the superior vena cava [2]. This type of central venous catheters (CVC) allows for easy access to a patient’s circulation, incur minimal restriction in normal activities and usually at a low risk of complications [3]. Next to VAPs, other methods have also been introduced, such as the Hickman catheter and peripherally inserted CVC (PICC) lines [4, 5]. However, all devices constitute some risk of venous access related adverse events (VARAE).

As anticancer drug, trabectedin stands out as a drug with a unique mechanism of action, having effect both at the level of tumor DNA and on the tumor microenvironment [6]. It is one of the few drugs active in STS [7]. The drug has a manageable toxicity profile, but life-threatening toxicity due to uncommon adverse events has been reported [8]. Thus far several papers have mentioned VARAE, including reports on trabectedin extravasation and associated thrombi on the line tip, but no papers focusing on VARAE in detail have been published [9–12]. This article aims to systematically study VARAE observed in 127 consecutive sarcoma patients treated with trabectedin and to evaluate the measures taken to handle these problems.

## Methods

A retrospective analysis of VARAE in all patients treated with trabectedin was performed in all five participating Dutch sarcoma referral centers: the Leiden University Medical Center (LUMC), the Netherlands Cancer Institute Antoni van Leeuwenhoek (NKI-AvL), the Erasmus MC Cancer Institute (EMC), the Radboud University Medical Center (RUMC) and the University Medical Center Groningen (UMCG). Patients were eligible when treated with trabectedin monotherapy for advanced STS. Data on patient characteristics were reported as well as the type of venous access device, its placement, adverse events related to its usage and the interventions to counter these events. Adverse events related to the VAD placement were ignored, as these have no direct relation with trabectedin infusions. Hence, all events described occurred after at least one cycle of trabectedin had been given.

To test for a difference in the number of cycles per VAD, a Kruskal–Wallis non-parametric test was used. To assess differences between VARAE per VAD cross tables and the Chi square were computed. All statistical analyses were performed using SPSS version 20.

## Results

### Patients

In total, 127 advanced STS patients were treated with single agent trabectedin between November 1999 and November 2014. Almost all patients were treated as part of an observational phase IV study or of the TRUSTS trial [13, 14]. Due to the inclusion criteria of these studies, trabectedin was given either as first line (15.0%), second line (59.1%), third line (16.5%), fourth line (7.1%) or as a further line of treatment (2.4%). The trabectedin treatment regimen was given at a dosage of 1.5 mg/m^2^ as 24 h infusion every three weeks in 89.8% of patients, the remaining patients received a lower dose (1.1–1.3 mg/m^2^) and/or a 3 h infusion. The most prevalent types of STS histology were leiomyosarcoma (40.9%), liposarcoma (26.0%) and synovial sarcoma (12.6%), as shown in Table 1.

**Table 1:** Patient characteristics

	N (%)
Sex	
Female	66 (52.0)
Male	61 (48.0)
Age	
Median (years)	54.3
Range (years)	25.6–79.5
WHO performance score	
0	52 (40.9)
1	66 (52.0)
2	9 (7.1)
Histology	
Leiomyosarcoma	52 (40.9)
Liposarcoma	33 (26.0)
Synovial sarcoma	16 (12.6)
Various others	26 (20.5)
Best response	
Partial response	8 (6.3)
Stable disease	64 (50.4)
Progressive disease	45 (35.4)
Not evaluable	10 (7.9)
Hospital	
LUMC	48 (37.8)
NKI-AvL	40 (31.5)
EMC	15 (11.8)
RUMC	12 (9.4)
UMCG	12 (9.4)

### VADs inserted

The VAP was used in 102 (80.3%) patients, of which 87 were identified as a Smith Medical Port-a-Cath^®^. Hickman catheters and PICC lines were inserted in 15 (11.8%) and 10 (7.9%) of patients, respectively. A total of 540 cycles of trabectedin were given with a median number of 4 cycles for the entire patient group. The number of cycles given did not differ significantly per VAD (data not shown).

Each hospital had a clear preference for a particular type of VAD that was initially inserted; in the LUMC VAPs (100% of patients), in the NKI-AvL VAPs (95%), in the EMC the Hickman catheter (100%), in the RUMC a PICC line (66.7%) and in the UMCG VAPs (100%). VADs were inserted by a dedicated team of health care workers to ensure low incidence of complications related to the VAD placement.

Of all patients, only three patients with a Hickman catheter requested their VAD to be replaced by another type of VAD. Two of these patients preferred a VAP, but did not have a VARAE at the time of replacement. In another patient, the catheter was chronically obstructed due to a thrombus at the catheter tip, which required catheter flushing by a radiologist, despite adequate antithrombotic treatment.

### Sterile inflammation along the catheter trajectory

**Fig. 1:**
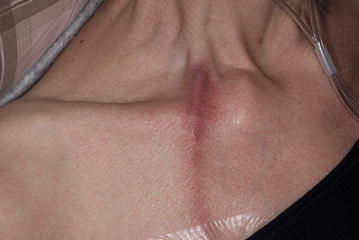
Typical sterile inflammation along the venous access port catheter trajectory

Out of the 127 patients, 45 patients (35.4%) with a VAP developed a varying degree of inflammation along the catheter trajectory, which could include erythema, pain or swelling, as shown in Fig. 1. In between cycles these symptoms waned, but a few days after the following infusion a flare up was often noted. Fever was neither reported by patients, nor observed during physical examination at admission or at the outpatient clinic. The skin surrounding the port’s reservoir was not affected and the VAD could be used for infusions normally. Bacterial cultures could not identify an etiological micro-organism for these symptoms in all, but three patients.

In the first instances these symptoms were deemed a result of cellulitis and oral antibiotics were prescribed (flucloxacillin 500 mg four times daily). However, the symptoms abated only mildly and the erythema remained unchanged for weeks and existed even after the discontinuation of trabectedin therapy. Extra intravenous infusion of normal saline fluids during trabectedin infusion appeared to ease the symptoms, especially the pain.

**Fig. 2:**
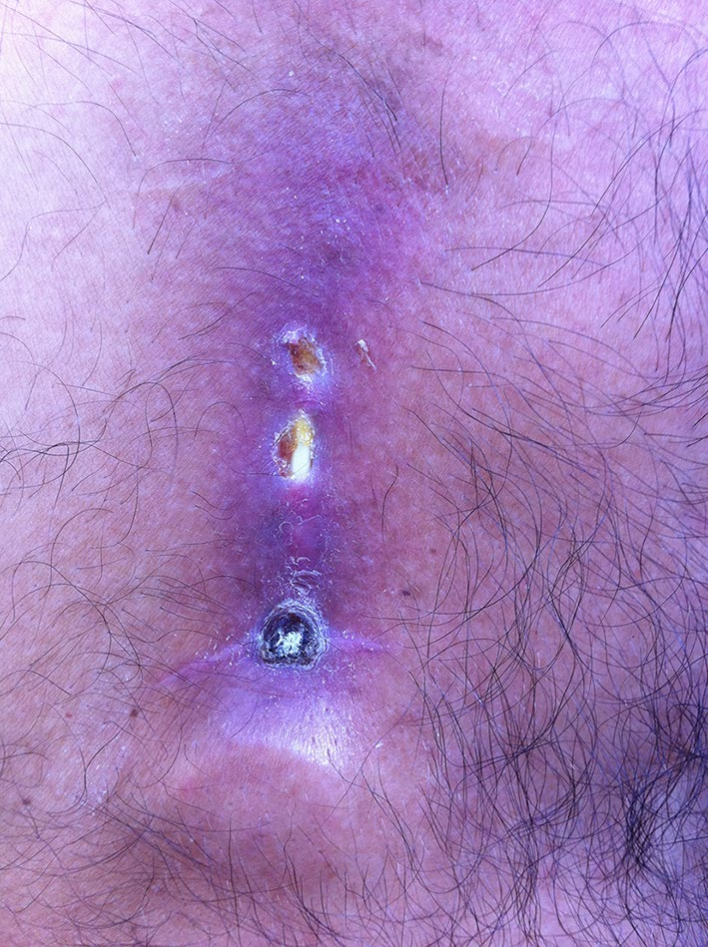
Skin erosion along venous access port catheter trajectory due to severe sterile inflammation

In a single patient with port VAD, the inflammation became rampant and in the course of several weeks it led to severe skin erosion along the catheter trajectory, as shown in Fig. 2. At progression of the inflammatory aspect of the skin, the patient was treated with oral antibiotics. Due to the skin destruction, a local secondary cellulitis developed. Despite this, the patient did not feel ill. As the patient did not show symptoms of acute infection, it was decided to continue trabectedin treatment. After trabectedin was stopped due to progressive disease, the VAP remained in place and was used for dacarbazine cycles without VARAE.

Remarkably, this complication appeared only in patients from one hospital and only after receiving several trabectedin cycles, and did not occur with any other type of cytostatic agent. As the same brand and type of VAD was used in another hospital without this complication, the dedicated teams compared their respective methods of VAD insertion. The only apparent difference found, was in the depth of the subcutaneous insertion for tunneling the catheter. Catheter insertion can be performed more or less deeper under the skin and the latter method was associated with the sterile inflammation along the catheter trajectory. Upon changing the local protocol to deepen the tunneling of the catheter, no further events of sterile inflammation of the catheter trajectory were observed.

### Adverse events related to VAD

All types of VADs used had VARAE, as shown in Table 2. For the whole patient cohort, the most common adverse events were erythema (30.7%) and pain (28.3%) at the VAD site or along the catheter trajectory. In 11.8% of patients these symptoms were diagnosed as an inflammation and/or infection, where inflammation consisted of swelling, painfulness and erythema. Blood cultures did not grow pathogenic micro-organisms. In some of these patients the ‘infection’ diagnosis could retrospectively be reclassified as the previously described sterile inflammation with near certainty. Several patients (11.0%) had a thrombus at the catheter tip at one or several instances. Often, these thrombi could be flushed with urokinase solution before proceeding to administer the trabectedin infusion without further complications. However, catheter thrombosis could also lead to VAD impairment. Remarkably, all of these patients were treated in the same hospital, which was also the hospital were VAPs were inserted with tunneled catheters deep in the epidermis. Thrombosis at the catheter tip and the sterile inflammation were not significantly associated (data not shown). The skin erosion and extravasation of trabectedin were seldom seen. Dislocation or pinch-off was not seen in any patient. Due to the small number of patients with a Hickman catheter or PICC line, no statistical differences in the incidence of VARAE could be detected. Only a single patient (0.8%), who had a Hickman catheter, had an extravasation.

**Table 2:** Adverse events at VAD site/trajectory per venous access device

N (%)	Inflammation	Erythema	Pain	Infection	Thrombosis	Impairment	Erosion	Extravasation	All AE^a^
Venous access port (102)	35 (34.3)	30 (29.4)	28 (27.5)	9 (8.8)	11 (10.8)	5 (4.9)	1 (1.0)	0 (0.0)	44 (43.1)
Hickman line (15)	5 (33.3)	4 (26.7)	4 (26.7)	3 (20.0)	1 (6.7)	3 (20.0)	0 (0.0)	1 (6.7)	7 (46.7)
PICC line (10)	5 (50.0)	5 (50.0)	4 (40.0)	3 (30.0)	2 (20.0)	1 (10.0)	0 (0.0)	0 (0.0)	5 (50.0)
Total	45 (35.4)	39 (30.7)	36 (28.3)	15 (11.8)	14 (11.0)	9 (7.1)	1 (0.8)	1 (0.8)	56 (44.1)

^a^Summary of all types of adverse events per venous access device

### Interventions for VARAE

Oral antibiotics were given in 17.3% of patients, most often flucloxacillin, as shown in Table 3. Some patients received a prescription for oral antibiotics to be taken in case VAD related symptoms worsened. Although this was not sufficient to stop the erythema along the catheter trajectory, it may have helped against a secondary infection. In 5 patients (3.9%) VAD an infection necessitated IV antibiotics (2 patients with a VAP, 3 patients with a PICC line). Due to the severity of symptoms or VAD impairment VAD replacement was needed in 15.0% of patients. Patients with a VAP usually had the same type of VAP inserted at the contralateral side, patients with a Hickman catheter or PICC line most often received a VAP. As the problem of the sterile inflammation and other VARAE were better understood and recognized, in due course a wait-and-see policy was applied in a considerable number of patients (13.4%). Despite frequent complaints of pain at the VAD site, analgesics were only needed in a minority of these patients.

**Table 3:** Interventions for VAD related adverse events per venous access device

N (%)	Antibiotics (oral)	VAD replaced	Wait-and-see	Analgesics	Urokinase (IV)	Antibiotics (IV)
Venous access port (102)	19 (18.6)	10 (9.8)	13 (12.7)	8 (7.8)	8 (7.8)	2 (2.0)
Hickman line (15)	1 (6.7)	6 (40.0)	3 (20.0)	2 (13.3)	1 (6.7)	0 (0.0)
PICC line (10)	2 (20.0)	3 (30.0)	1 (10.0)	0 (0.0)	0 (0.0)	3 (30.0)
Total	22 (17.3)	19 (15.0)	17 (13.4)	10 (7.9%)	9 (7.1)	5 (3.9)

## Discussion

Trabectedin is one of the proven active drugs in the treatment of soft tissue sarcoma and is given through a central venous catheter to avoid peripheral vein damage. As treatment continues until progressive disease or unacceptable toxicity, is it important to evaluate catheter related complications. The sterile inflammation along the catheter trajectory found in this study was an unexpected VARAE and was initially poorly understood. Erythema or pain is usually taken as a sign of skin infection and treated as such. However, there were no other signs of infection such as positive cultures, and the severity of the skin complications appeared to be related to the administration of trabectedin. In addition, the erythema was most prominent along the catheter trajectory, which made a porous catheter likely to be the cause. A direct effect of trabectedin on the tissue surrounding the catheter could cause the inflammation, but this catheter porosity implies that only a small quantity of trabectedin permeates. This small quantity leads to fewer symptoms compared to a full trabectedin extravasation, as has been reported in literature [11].

To investigate the hypothesis of catheter porosity, the manufacturer of trabectedin, PharmaMar, offered to test a used catheter. A VAP was available that was previously used in a patient who had received several cycles of trabectedin with symptoms of sterile inflammation alongside the catheter trajectory and from whom the VAP was removed because of disease progression. The objective of the test was to determine if trabectedin permeates from the internal surface to the outside of the catheter during a 24 h infusion. High-performance liquid chromatography with diode array detection (HPLC–DAD) and multi-syringe flow injection system (MS-FIA) methods were used for detection of trabectedin in the dextrose 5% solution the VAP was submerged in. Neither test could detect trabectedin in samples taken from the dextrose 5% solution, which ruled out gross catheter porosity (PharmaMar communication). In our view, however, this could not rule out sub lower-limit of quantification leakage.

Non-infectious inflammation of the VAD site of various severity was also reported by Hoicyk et al. in addition of thrombi at the catheter tip. It was hypothesized that increased resistance due to small thrombi may be associated with drug backspill [12]. In the current study, neither an association of sterile inflammation and thrombosis was found, nor was reduced flow through the catheter observed. Catheter thrombosis occurred in several patients, which was treated by flushing the catheter with an urokinase solution. Thrombosis prophylaxis was not initiated at the start of trabectedin therapy in any of the participating centres.

In the patient cohort only a small number of patients had PICC lines. A larger retrospective series of STS and ovarian cancer patients was reported by Martella and colleagues. Out of 45 patients with a PICC line receiving trabectedin a device dislocation was reported in two patients and an infection in another two. PICC line malfunction or VARAE requiring VAD removal did not occur [10]. This implies that PICC lines may have lower incidence of associated toxicity than our current cohort suggests. However, the number of VARAE in patients using a PORT was also lower. Due to the retrospective nature of this patient series, relative underreporting compared to our study may have occurred, as almost all patient in this cohort where treated as part of a clinical trial.

The usage of a disposable elastomeric pump to administer a 24-h trabectedin infusion has been described [9]. Patients could choose for a regular VAP or a Baxter LV10 Pump which allowed patients to spend the night at home. Out of 28 patients 21 chose the ambulatory pump. This method was considered feasible and safe. However, most patients will receive trabectedin trough conventional VAPs reported on in this paper, and no data is available comparing these different techniques.

Compared to published safety data, the rate of observed trabectedin extravasation of 0.8% in our series was similar to 0.5% reported in large pooled analysis of 1132 patients who received single agent trabectedin [8].

## Conclusions

Despite the use of central venous access devices, trabectedin can cause local sterile inflammation along the catheter trajectory, in particular in venous access ports. Positioning the port’s catheter deeper in the subcutis appears to be the most efficient way to prevent this complication.

## Abbreviations

STS: soft tissue sarcomas
VAD: venous access devices (any type)
PICC line: peripherally inserted CVC line
CVC: central venous catheter
VAP: venous access ports (such as a Port-a-Cath)
VARAE: venous access related adverse events
